# Real-time stress and strain monitoring at the bedside: new frontiers in mechanical ventilation

**DOI:** 10.3389/fmed.2025.1692488

**Published:** 2025-10-06

**Authors:** Tommaso Pettenuzzo, Francesco Zarantonello, Alessandro Zambianchi, Gianluca Lumetti, Domenico Ruggerini, Giorgia Pacchiarini, Elisa Pistollato, Valentina Fincati, Giulia Mormando, Alessandro De Cassai, Nicolò Sella, Annalisa Boscolo, Paolo Navalesi

**Affiliations:** ^1^Department of Medicine, University of Padua, Padua, Italy; ^2^Institute of Anesthesia and Intensive Care, University Hospital of Padua, Padua, Italy; ^3^Department of Cardiac, Thoracic, Vascular Sciences, and Public Health, University of Padua, Padua, Italy

**Keywords:** ventilator-induced lung injury, overdistension, lung stress, lung strain, electrical impedance tomography, lung ultrasound, respiratory mechanics, pendelluft

## Abstract

Mechanical ventilation is a fundamental intervention in intensive care medicine, providing vital support for patients with severe respiratory failure. However, this life-sustaining therapy also carries the risk of harm. Ventilator-induced lung injury (VILI) is now predominantly understood in terms of lung overdistension, characterized by excessive stress and strain on pulmonary tissue. In recent years, a variety of novel monitoring strategies have emerged, from refined measurements of respiratory mechanics to advanced imaging and physiologic modeling, to help in bedside detection of excessive lung stress and strain. Electrical impedance tomography is a non-invasive tool providing real-time imaging of regional ventilation and assisting in the diagnosis of overdistension and its minimization through positive end-expiratory pressure titration, also during partial support ventilation. Pleural and lung ultrasound might also suggest the occurrence of overdistension, although clinical data are still preliminary. Bedside maneuvers, such as changing patient positioning or applying abdominal weights, can help identify overdistension by observing change in respiratory mechanics. Ventilator-based methods like the recruitment-to-inflation ratio and the overdistension index help assess the risk of overdistension, despite requiring careful interpretation and validation. Biomarkers such as Clara cell secretory protein-16 and stretch-induced gene signatures represent a promising avenue for real-time monitoring of lung injury, though further validation is needed. These tools aim to help clinicians individualize ventilator settings, balancing adequate gas exchange with lung protection. Despite this progress, most techniques remain in the realm of research. Few have undergone the rigorous physiological and clinical validation necessary for routine bedside use. As the critical care community moves toward more personalized ventilation strategies, establishing reliable, real-time methods to assess lung stress and strain at the bedside will be key to translating innovation into improved patient outcomes.

## Introduction

The notion that mechanical ventilation can be harmful dates back centuries. In 1744, John Fothergill described the successful resuscitation of an apneic and pulseless coal miner by William Tossach, who used mouth-to-mouth ventilation to inflate the patient’s lungs and restore a heartbeat. Fothergill also expressed concern that mechanical bellows might cause more harm than manual insufflation, as the force delivered by bellows could not be regulated as precisely as a human breath. This early recognition of potential harm from mechanical ventilation foreshadowed the modern concept of ventilator-induced lung injury (VILI) (1).

In the 1940s, Macklin and Macklin demonstrated that elevated airway pressures could rupture alveolar walls, allowing air to dissect into surrounding tissues, an observation that contributed to the definition of “barotrauma” (1). In the 1960s, growing concern over oxygen toxicity, particularly based on studies in animals and neonates, led clinicians to favor high tidal volumes (Vt) as a means of improving oxygenation while avoiding the risks associated with high fractions of inspired oxygen (FiO₂) (1). As a result, in the early era of modern mechanical ventilation, barotrauma became a frequent complication, spurring significant research into its pathophysiology.

A pivotal 1974 study by Webb and Tierney demonstrated in animal models that high distending pressures could cause fatal pulmonary edema, highlighting the risks of excessive lung stretch (2). In 1988, Dreyfuss and colleagues showed that lung injury was primarily due to excessive end-inspiratory lung volume rather than airway pressure alone, laying the foundation for the concept of “volutrauma” (3).

The distinction between barotrauma and volutrauma is theoretical, as lung overdistension results from any Vt that generates an excessive transpulmonary pressure, i.e., the transmural pressure gradient across the alveolar wall (4). The primary mechanical determinants of lung overdistension for a given Vt are lung compliance, airway pressure variation and the pressure generated by inspiratory muscles (5). Lower lung compliance implies a smaller aerated lung volume, leading to greater dynamic lung strain, defined as the ratio of Vt to resting lung volume (6). Additionally, reduced lung compliance lowers the ratio of lung to total respiratory system compliance, resulting in higher end-inspiratory transpulmonary pressure (lung stress) for a given airway pressure (4). Finally, stronger inspiratory efforts in patients receiving partial ventilatory support also increase end-inspiratory transpulmonary pressure by generating excessively negative pleural pressure (5).

Several studies provide strong evidence that VILI increases mortality in patients receiving controlled mandatory ventilation (7). Therefore, timely and accurate assessment of lung stress and strain is key to improving patients’ outcomes. Each of the static components of the respiratory cycle, namely tidal volume (Vt), plateau pressure (Pplat), and positive end-expiratory pressure (PEEP), may require adjustment in order to mitigate the risk of lung overdistension. The individual patient’s response to these ventilator settings should be evaluated to balance their benefits and potential side effects. For example, PEEP may be beneficial in patients with adequate potential for lung recruitment, reducing atelectrauma (injury related to the repetitive opening and closing of alveolar units), while minimizing dynamic strain and improving ventilation-perfusion matching and oxygenation. Conversely, in patients with limited recruitment potential, high PEEP levels may lead to overdistension, increased dead space, and hemodynamic compromise (8).

Over the years, various techniques have been proposed to estimate lung stress and strain at the bedside, including assessment of breathing patterns and respiratory mechanics (e.g., Vt, Pplat, driving pressure [ΔP], transpulmonary pressure [Ptp], stress index [SI], C20/C ratio) (7, 9–11), as well as chest computed tomography (CT) (12). Recently, several innovative methods have emerged as promising tools to estimate the risk of lung overdistension. Imaging modalities, such as pleural and lung ultrasound (LUS) and electrical impedance tomography (EIT), have shown growing utility. Novel applications of respiratory mechanics, assessed directly at the ventilator following specific changes in ventilator settings or patient positioning, may also provide valuable insights.

The aim of this narrative review is to present emerging techniques that, over the last few years, have shown potential to support critical care physicians in identifying non-physiologic stress and strain at the bedside (Table 1). As such, we will not discuss other mechanisms of VILI, such as atelectrauma and ergotrauma (injury resulting from mechanical energy delivered to the respiratory system during each breath) (13), nor will we address the hemodynamic effects of non-physiologic stress and strain (5).

**Table 1:** New frontiers in bedside assessment of lung stress and strain.

Technique	Rationale for overdistension management	Advantages	Disadvantages
EIT	Analyzes the temporal and spatial variations of thoracic impedance, used as surrogate of variations of ventilation and perfusion	Non-invasive, radiation-free, bedside. Real time assessment of ventilation and perfusion.	Not widely available. Requires dedicated equipment and technical expertise. Limited spatial resolution. Explores a limited portion of the lung. Influenced by the position of the electrodes. Contraindicated during pregnancy and in patients with electronic devices, skin lesions or burns.
Overdistension-collapse method	Assesses variations in pixel compliance during a decremental PEEP trial and calculates the cumulative compliance losses for higher and lower PEEP levels *PEEP may be titrated to a desired percentage of overdistension*	Identify the amount of overdistension for every PEEP level	Assumes that extreme PEEP levels are associated with 0% collapse and overdistension. The crossing point between overdistension and collapse varies based on the explored PEEP range and patient’s position. May overestimate overdistension, compared to CT.
Fraction of ventilation	Calculated as the TIV in the ventral or dorsal half of the lung divided by the TIV in the whole lung x 100 *PEEP may be titrated to avoid ventral hypoventilation, suggestive of overdistension*	Classifies phenotypes of ventro-dorsal ventilation distribution	Less reliable for focal lung diseases. Requires ROI selection.
Center of ventilation	Defined as the vertical coordinate that marks the point where the sum of ventral and dorsal ventilation divides the lung into two equal parts *PEEP may be titrated to CoV ≤ 50%, avoiding overdistension of the non-dependent lung and a shift in ventilation to the dependent lung*	Avoids the need for ROI selection	Cannot detect differences between the left and right lung in asymmetrical lung injury
Silent spaces	Regions with TIV < 10% of maximal TIV *PEEP may be titrated to decreased silent spaces in regions at risk of overdistension*	Does not require measurement of regional compliance	Need devices applying lung contouring
Regional peak flow	Defined as the maximum regional peak flow for all aerated pixels at a specific PEEP level *PEEP may be titrated to a desired percentage of overdistension, calculated based on the RPF*	Applicable during spontaneous breathing	Assumes that RPF explains regional pulmonary compliance. Further physiological and clinical data are needed.
Overdistension-collapse method based on dynamic lung compliance	Assesses variations in dynamic pixel compliance (pixel TIV/dynamic transpulmonary driving pressure) during a decremental PEEP trial *PEEP may be titrated to a desired percentage of overdistension*	Applicable during spontaneous breathing	Further physiological and clinical data are needed
Pleural and lung ultrasound		Non-invasive, easy-to-use, bedside. Provides valuable information on global and regional lung aeration and PEEP-induced recruitment.	Operator-dependent, requires technical expertise. Sensitivity can be reduced by subcutaneous emphysema or large thoracic dressings. Low sensitivity for diseases with no or minimal extension to peripheral fields. No specific lung ultrasound sign for the detection of lung overinflation.
Maximal longitudinal pleural strain	Estimates the deformation of the pleural line over time by following the motion of speckles created by ultrasound beam scattering through tissue *PEEP may be titrated to a desired percentage of overdistension*	Might estimate the amount of overdistension	Requires device and software for strain analysis. Further physiological and clinical data are needed.
Patient positioning	Abdominal weight *Improved respiratory mechanics abdominal weight may indicate excessive PEEP*	Non-invasive, easy-to-use, bedside	Effect of weight needs to be studied on the long term and with larger sample sizes. Contraindicated in case of surgical incisions, recent abdominal wounds, severe abdominal hypertension. Unclear technical details (best site for chest wall loading, ideal pressure, methods of pressure application).
	Supine-flat position *The improved respiratory mechanics with supine-flat position may indicate excessive PEEP*	Non-invasive, easy-to-use, bedside	Effect of supine-flat position needs to be studied on the long term and with larger sample sizes. Supine-flat position may determine increased ventilation inhomogeneity, reduced EELV, and increased lung strain. May increase risk of ventilator-associated pneumonia.
R/I ratio	PEEP reduction by 10 cmH_2_O to calculate the ratio of the compliance of the recruited volume at high PEEP to the compliance at low PEEP (after accounting for airway closure) *For R/I ratio <0.3–0.4, setting a lower PEEP is advisable, while for R/I ratio > 0.6–0.7, a higher* *PEEP can be considered*	Non-invasive, easy-to-use, bedside. Correlates well with PEEP-induced changes in lung strain and with the percentage of lung overdistension in patients with COVID-19-related ARDS. May help identify PEEP responsiveness during general anesthesia and prone positioning.	Common clinically relevant underestimations or overestimations with modern ICU ventilators. Reliance on a single cut-off to guide treatment potentially inappropriate. Lung strain and compliance not uniform in the conventionally tested 10-cmH_2_O PEEP range. Poor diagnostic performance to predict lung recruitability, when compared with CT scan.
Overdistension index	Pressure difference between the flattened breath and non-flattened breath on a pressure-volume loop *Safe range of 0–0.8 cmH_2_O (when calibrated to the SI)*	Non-invasive and real-time. Extensible to ventilation modes different from VCV.	Evidence limited by small sample size and direct physiological correlation with overdistension. Needs to be tested for ventilator modes different from VCV.
Biomarkers	Molecules released during the local and systemic inflammatory response associated with ventilator-induced lung injury	Might offer the opportunity for a personalized, biologically-informed approach to prevent lung overdistension	May need long laboratory turnaround times. Further physiological and clinical data are needed.

EIT, electrical impedance tomography; PEEP, positive end-expiratory pressure; CT, computed tomography; TIV, tidal impedance variation; ROI, region of interest; CoV, center of ventilation; RPF, regional peak flow; R/I, recruitment-to-inflation; COVID-19, coronavirus disease-19; ARDS, acute respiratory distress syndrome; VCV, volume-controlled ventilation. References are provided in the main manuscript.

## Electrical impedance tomography

EIT is a non-invasive, radiation-free, and validated bedside technique for real-time, continuous evaluation of the regional distribution of ventilation and perfusion (14). EIT analyzes the temporal and spatial variations in thoracic impedance in response to very small alternating electrical currents applied through pairs of electrodes embedded in a belt wrapped around the patient’s chest (14). Impedance variations during air and blood movement are used as surrogates for ventilation and perfusion, respectively (14).

Multiple applications of EIT in both invasive and non-invasive mechanical ventilation have been described (15). One of the most common is PEEP titration, which is critical for enhancing the physiological benefits, while minimizing the potential adverse effects, of PEEP. During a decremental PEEP trial, regional compliance changes are estimated pixel by pixel as the ratio of tidal impedance variation (TIV), defined as the amplitude of impedance changes between end-expiration and end-inspiration, to ΔP, calculated as the difference between Pplat and PEEP (16). Compliance losses at higher and lower PEEP levels are interpreted as overdistension and collapse, respectively, and summed across all pixels in the lung region where the belt is positioned. The optimal PEEP is typically defined as the level corresponding to the intersection point of the cumulative compliance loss curves across the explored PEEP range, representing the minimal difference between the percentages of overdistension and collapse (16). This “overdistension-collapse method” has been primarily applied for PEEP selection during continuous mandatory ventilation (15). Despite known limitations (Table 1), this technique offers a unique opportunity to quantify and monitor the regional extent of overdistension and to adjust PEEP to target a desired level.

Alternative EIT-based approaches for PEEP titration aiming at minimizing overdistension assess changes in the spatial distribution of TIV. A decrease in TIV, especially in non-dependent lung regions, after a PEEP increase, may indicate regional overdistension (17). These distributional ventilation changes can be quantified using the dorsal-to-global or ventral-to-global ventilation fractions, calculated as the proportion of ventilation occurring in the dorsal or ventral regions, respectively, relative to total ventilation detected in the image. In a cohort of 128 postoperative patients at high risk for pulmonary complications, a ventilation pattern characterized by dorsal predominance and ventral hypoventilation, suggestive of ventral overdistension, was associated with higher complication rates and delayed oxygen weaning (18, 19).

PEEP can also be titrated by assessing changes in compliance across four horizontal lung regions following a PEEP increase, to evaluate recruitability, and ΔP reduction, to evaluate alveolar cycling and overdistension (20). In 20 patients with ARDS, this EIT-based protocol led to improved oxygenation and reduced alveolar cycling without promoting global overdistension (20).

The ventro-dorsal center of ventilation (CoV) represents the weighted geometric center of the ventilation distribution (21). A CoV greater than 50% indicates predominant dependent ventilation and may suggest overinflation of the non-dependent (ventral) lung, thereby assisting in the identification of excessive PEEP (22).

An increase in “silent spaces,” defined as those pixels exhibiting less than a specified percentage of the maximum TIV, within regions at risk for overdistension (e.g., above the CoV) may support the decision to reduce PEEP (23–25). In 43 patients with coronavirus disease-19 (COVID)-associated acute respiratory distress syndrome (ARDS), Taenaka et al. found that, among patients with high lung recruitability, the combination of high PEEP and prone positioning achieved the best oxygenation and minimized dependent silent spaces (indicative of potential collapse), without increasing non-dependent silent spaces (indicative of overdistension). In contrast, among low recruiters, high PEEP, compared to low PEEP, increased non-dependent silent spaces, in both supine and prone positions (25).

EIT also allows quantitative estimations of the overall degree of spatial ventilation heterogeneity. The global inhomogeneity index (GI) is calculated as the sum of the differences between each pixel’s TIV and the median TIV, normalized by the total TIV within the lung area (26). In a series of 14 patients with ARDS and chronic obstructive pulmonary disease, a PEEP titration strategy based on the minimum GI index value, compared to the ARDS Network lower PEEP/FiO_2_ table, improved the ventilatory ratio, reduced mechanical power, and enhanced cardiac index and oxygen delivery (27). However, some reports found this strategy to be associated with higher ΔP and overdistension, compared to other methods of PEEP titration (28).

Finally, EIT enables detection of regional aeration changes and quantification of regional strain (29) and may aid in identifying regional overdistension (30) and predicting PEEP responsiveness (31).

Although EIT is not yet widely available, requires technical expertise, and presents some contraindications and limitations (14) and although many EIT-derived techniques for assessing overdistension still lack physiological and clinical validation, EIT remains a promising tool to complement other strategies for the prevention and diagnosis of lung overdistension (Table 1). Preliminary clinical evidence suggests that EIT-guided PEEP titration may be associated with improved respiratory system mechanics and patient survival (32).

## Pleural and lung ultrasound

Pleural and lung ultrasound (LUS) is a non-invasive, easy-to-use bedside tool that, despite known limitations (33), provides valuable information not only on global and regional lung aeration but also on PEEP-induced recruitment (34). Some experts have suggested that reduced lung sliding in non-dependent regions may be indicative of lung overdistension (35). However, clinical evidence remains very limited.

Tonelotto et al. observed that, in 18 patients with healthy lungs under general anesthesia, the presence of six or more horizontal reverberation lines may indicate lung overdistension, as evaluated by EIT (36). Preliminary clinical data from pilot studies have explored the use of pleural strain measurement, defined as the deformation of the pleural line over time, for this purpose. In a study involving 10 patients undergoing general anesthesia for elective surgery, Girard et al. demonstrated the feasibility and reproducibility of pleural strain measurement using ultrasound elastography, which showed significant correlations with Vt (37). Maximal longitudinal pleural strain (MLPS), assessed by speckle tracking, an ultrasound technique following the motion of speckles created by ultrasound beam scattering through tissue, has also been suggested for this purpose. In a recent study of 30 patients with acute hypoxemic respiratory failure, Persona et al. found a significant correlation between MLPS and PEEP-induced overdistension, as estimated by EIT. Specifically, an MLPS value <10 was associated with EIT-derived overdistension ≥10%, while values <7 were indicative of severe overdistension (≥30%) (38).

These findings are extremely preliminary and warrant confirmation in larger patient cohorts, as well as validation using other techniques, such as chest CT, before LUS can be considered a reliable tool for assessing lung overdistension (Table 1).

## Bedside maneuvers

In addition to instrumental techniques, several bedside maneuvers have been proposed to offer clinically useful insights into lung overdistension (Table 1). Recent studies, primarily involving patients with COVID-19-related ARDS, have reported that applying an external weight to the sternum or abdomen paradoxically reduces airway pressure, Pplat, and ΔP, while increasing respiratory system compliance, contrary to expected physiological responses (39). This counterintuitive phenomenon has been termed the “mechanical paradox” (40).

In 2025, Pacchiarini et al. conducted a prospective interventional study to investigate the underlying mechanisms of this paradox (39). Twenty patients receiving invasive mechanical ventilation for acute hypoxemic respiratory failure were enrolled and studied during a decremental PEEP trial under EIT and esophageal pressure monitoring. Variable external weights were applied to the abdomen to induce a 5-mmHg increase in intra-abdominal pressure. At each PEEP level, three sequential phases were performed: weight-off, weight-on, and weight-off. The study demonstrated that the physiological response to abdominal loading depended on whether lung overdistension or collapse was predominant. In cases where PEEP-induced overdistension prevailed, i.e., PEEP levels above the EIT-defined optimal PEEP according the overdistension-collapse method, abdominal loading led to a decrease in peak, plateau, and driving pressures and an increase in both lung and respiratory system compliance. These effects were accompanied by a reduction in end-expiratory lung impedance (EELI), likely reflecting a decrease in end-expiratory lung volume (EELV). The effect of the abdominal weight needs to be studied over the long term and in larger sample sizes and the optimal pressure to apply, as well as the method of application, remains unclear. However, these findings suggest that abdominal loading may serve as a simple bedside maneuver to identify overdistension and assist in PEEP titration. Specifically, a decrease in peak, plateau, and driving pressures in response to abdominal weight application may indicate excessive PEEP, which should be adjusted downward.

Patient positioning is also a critical determinant of respiratory mechanics in both the operating room and the intensive care unit (ICU). Recent evidence indicates that certain positions may influence the risk of overdistension. Pearce et al. evaluated the effects of supine versus semi-recumbent positioning in 14 mechanically ventilated patients with ARDS (41). Using EIT and standard respiratory mechanics, they showed that moving from a semi-recumbent (35–40°) to a supine-flat position significantly improved respiratory system compliance and reduced ΔP. This positional change was also associated with a mild ventral redistribution of ventilation, potentially relieving ventral overdistension caused by gravitational pleural pressure gradients from the lung apex to base (41).

Similar findings were reported by Marrazzo et al. in two studies on patients with COVID-19-associated ARDS. In the first study, reducing trunk inclination from 40° to 0° significantly decreased end-inspiratory Ptp and increased lung compliance (42). The second study confirmed these results and further demonstrated improved ventral regional compliance and redistribution of tidal ventilation toward ventral regions in the supine-flat position. These improvements were attributed primarily to reduced lung overdistension, as also reflected by an improved ventilatory ratio (43).

Additionally, Bouchant et al. studied 30 patients with ARDS undergoing progressive trunk elevation (from 30° semi-seated to 0°, 30°, 60°, and 90° verticalization without body flexion). They found that both transpulmonary ΔP and EELV, as estimated using the nitrogen wash-in/wash-out method, increased progressively up to the straight 60° position, while lung strain decreased until the straight 30° position (44). These results suggest that verticalizing the patient from 0° to 60° may promote alveolar recruitment but at the cost of increased overdistension and reduced ventilator efficiency.

Overall, these findings support the potential utility of a simple and widely applicable maneuver, i.e., placing the patient in a supine-flat position, to help identify lung overdistension. Such a maneuver may prompt re-evaluation of ventilator settings, such as PEEP, once the patient is returned to the semi-recumbent position, which remains the recommended posture due to its association with a lower risk of ventilator-associated pneumonia (44).

## Mechanical ventilator maneuvers

Some recently described mechanical ventilator maneuvers may aid in assessing the risk of lung overdistension during invasive mechanical ventilation (Table 1).

Chen et al. validated a single-breath bedside method to estimate lung recruitability in patients with ARDS, including those with airway closure. Forty-five patients underwent an abrupt PEEP reduction from 15 to 5 cmH_2_O, and the recruitment-to-inflation (R/I) ratio was calculated as the ratio of the compliance of the recruited volume at high PEEP to the compliance at low PEEP. Based on the median R/I ratio of the study population, patients were classified as high recruiters (R/I ratio ≥ 0.5) or low recruiters (R/I ratio < 0.5). In 41 patients with airway closure below 15 cmH_2_O, the recruited volume estimated by this single-breath method correlated strongly with the reference pressure-volume curve method (45).

Despite several limitations (Table 1), the R/I ratio is a promising tool for bedside assessment of PEEP-related protection against lung overdistension. It has been shown to correlate well with PEEP-induced changes in lung strain (46, 47). Furthermore, it demonstrated a strong inverse correlation with the percentage of lung overdistension, as measured by EIT, in patients with COVID-19-related ARDS (25, 48). A weaker but still significant correlation was also observed in patients undergoing ultraprotective ventilation during veno-venous extracorporeal membrane oxygenation support (49). Moreover, the R/I ratio may help identify PEEP responsiveness during general anesthesia and prone positioning (50, 51).

Recruitment maneuvers (RMs), although potentially beneficial for reopening collapsed alveoli, may induce overdistension in already well-aerated regions, leading to clinically evident barotrauma, worsened ventilation-perfusion mismatch, and hemodynamic compromise (52). Recently, Santarisi et al. performed a post-hoc analysis of the EPVent2 trial, a multicenter, randomized, phase II study comparing a Ptp-guided ventilation strategy to a high-PEEP strategy for improving 28-day mortality and ventilator-free days in patients with ARDS (53). The authors examined the dose–response relationship between end-inspiratory Ptp after RM and subsequent changes in lung elastance as a surrogate of overdistension. Their results demonstrated that higher end-inspiratory Ptp were significantly associated with increased likelihood of elevated lung elastance, supporting the use of Ptp as a physiologic target to avoid overdistension during RMs (54). Additionally, patient-specific characteristics, markers of disease severity, and ventilatory parameters were identified as predictors of elevated Ptp during RMs, suggesting the potential for personalized optimization of recruitment strategies (54).

Another parameter for assessing overdistension is the stress index (SI), derived from the shape of the airway pressure–time waveform during volume-controlled ventilation. A concave upward shape (SI greater than 1.1) suggests overdistension, whereas the non-injurious SI range is considered to be 0.95–1.05 (10, 55, 56). However, SI is limited to volume-controlled ventilation, while a broader range of modes is commonly used in clinical practice. To overcome these limitations, Sun et al. proposed a novel overdistension index (OD), based on analysis of the pressure–volume loop. As peak inspiratory pressure increases, flattening of the end-inspiratory segment of the pressure–volume loop may indicate potential overdistension. The OD is calculated as the pressure difference between the end-inspiration of a “flattened” breath and the expected pressure of a “non-flattened” breath. In a cohort of 19 patients with ARDS, OD strongly correlated with SI, especially in those with moderate-to-severe ARDS. Calibration of OD to SI identified a potentially safe OD range of 0–0.8 cmH_2_O (57). Although these results are preliminary and not yet validated against direct physiological evidence of overdistension, they suggest that OD could help detect overdistension across a broader range of ventilation modes.

## Biomarkers

One of the emerging frontiers in the assessment of lung overdistension during mechanical ventilation is the use of molecular biomarkers. Structural lung damage associated with VILI can trigger a local and systemic inflammatory response, termed “biotrauma,” mediated by mechano-transduction intracellular pathways, which may contribute to the development of multiorgan failure (58). Key biomarkers implicated in biotrauma include tumor necrosis factor-alpha (TNF-*α*), interleukins 6 and 8 (IL-6, IL-8), surfactant protein-D (SP-D), and Clara cell secretory protein-16 (CC-16). Notably, CC-16 is a lung-specific protein secreted by epithelial cells in the small airways and has been strongly associated with lung overdistension (59).

Several clinical studies have reported associations between plasma or bronchoalveolar lavage (BAL) biomarker levels and mechanical ventilation strategies involving higher versus lower PEEP and/or Vt (60). Recently, López-Martínez et al. conducted sequential pooled transcriptomic analyzes to identify micro-ribonucleic acids and genes involved in the cellular response to cyclic stretch (61). These molecular signatures were subsequently validated in multiple experimental models, including *in vivo* animal models of stretch-induced lung injury, *ex vivo* human lung preparations, BAL samples from patients exposed to mechanical ventilation, and serum samples from mechanically ventilated patients with COVID-19. The authors identified two distinct transcriptomic signatures, comprising six micro-ribonucleic acids and six genes, respectively, that were capable of specifically detecting lung stretch (61).

Although further physiological and clinical validation is necessary, these findings offer a promising foundation for personalized, biologically-guided mechanical ventilation strategies aimed at preventing lung overdistension.

## Overdistension in the spontaneously breathing patient

Spontaneous breathing during mechanical ventilation has been associated with several advantages, including enhanced gas exchange and hemodynamics, reduced sedative requirements, and a decreased risk of diaphragmatic atrophy (62–64). However, growing evidence also highlights its potential to cause harm (65).

Patient self-inflicted lung injury (P-SILI) refers to lung damage resulting from excessive inspiratory efforts driven by elevated respiratory drive, particularly in patients with preexisting lung injury. Several mechanisms for P-SILI have been postulated, such as excessive transpulmonary pressure, pendelluft (gas shifts within the lung during inspiration, causing transient regional overdistension and localized injury), intra-tidal recruitment, local lung volutrauma, and negative pressure pulmonary edema (66).

Recognizing patients at risk for P-SILI is crucial, as unchecked respiratory effort can exacerbate lung injury. Various methods have been proposed over the years to assess this risk (66). Recent physiological studies in patients with COVID-19-related ARDS suggest that individuals receiving non-invasive ventilation who generate high inspiratory efforts, estimated by large negative deflections in esophageal pressure, and correspondingly elevated transpulmonary ΔP may face a higher risk of intubation (67, 68). Therefore, monitoring these physiological variables may be essential for the prevention and management of P-SILI. Nonetheless, additional clinical data are required to establish the utility of esophageal pressure monitoring during non-invasive ventilation.

Furthermore, strategies have been developed to enable the use of EIT during partial support ventilation by overcoming the challenges of PEEP titration in this setting, such as variable inspiratory effort, fluctuating compliance, and motion artifacts. The regional peak flow (RPF) method has recently been shown to be both feasible and comparable to the overdistension-collapse method (69, 70). RPF is calculated as the maximum first derivative of the inspiratory limb of the impedance signal. Relative changes in RPF, which are independent of a stable plateau pressure phase, are then used to estimate overdistension and collapse, similar to the overdistension-collapse approach (16), based on the assumption that regional peak airflow reflects regional pulmonary compliance (69). Additionally, EIT has been combined with dynamic Ptp measurements to apply the overdistension-collapse method during pressure support ventilation (PSV). In a study of 30 patients with ARDS on PSV, Mauri et al. found that titrating PEEP to the level minimizing the difference between the percentages of overdistension and collapse, as estimated via dynamic transpulmonary compliance, led to reduced respiratory drive and lower inspiratory effort, compared to PEEP titrated based on the lower PEEP-FiO_2_ table (71).

EIT has also been employed in the detection of pendelluft. In a retrospective observational study of 94 patients receiving PSV, pendelluft was detected in 41% of cases and was associated with a higher GI index, indicating more uneven ventilation distribution (72). Patients with pendelluft had fewer ventilator-free days at day 14 and longer ICU stays (72). In 108 difficult-to-wean ICU patients undergoing a T-piece spontaneous breathing trial (SBT), pendelluft was present in 70% and correlated with longer mechanical ventilation, fewer ventilator-free days, and significantly higher 28-day mortality (73). In another observational study of 20 patients undergoing sequential 2-cmH_2_O reductions in pressure support level during PSV, pendelluft volumes correlated positively with clinical markers of respiratory distress, including higher respiratory rate and P0.1 (74).

Finally, EIT has been recently employed to monitor the homogeneity of ventilation during SBTs. In a prospective observational study of 98 patients, Phoophiboon et al. observed that patients who were successfully weaned from mechanical ventilation (40 out of 98 patients) exhibited consistently smaller ventral-to-dorsal ventilation differences throughout the SBT, compared to those who failed the trial (75).

## Conclusion

**Figure 1:**
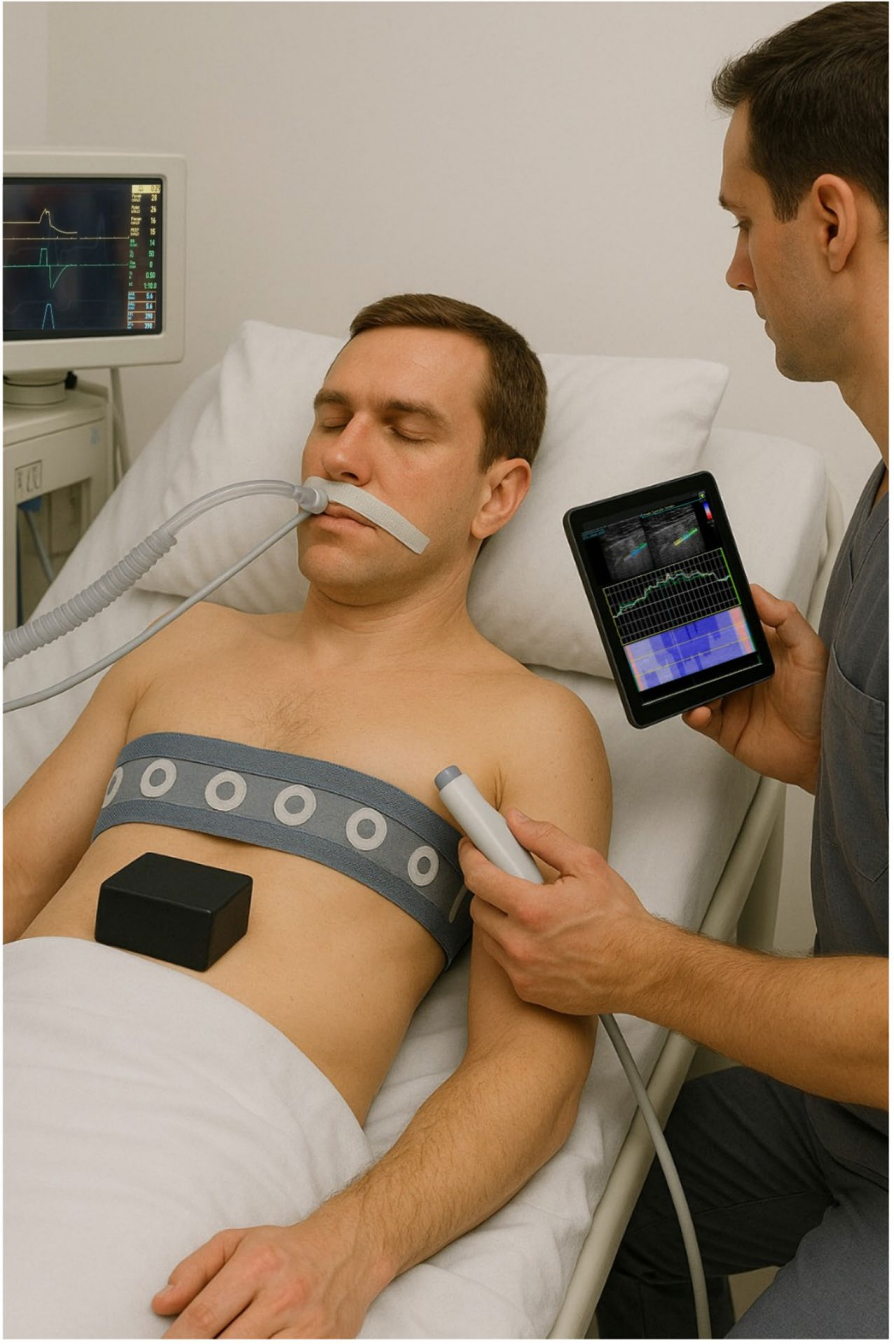
An expanding array of tools is enriching the future intensivist’s armamentarium for bedside detection of non-physiologic lung stress and strain. The physician is performing pleural and lung ultrasound to measure maximal longitudinal pleural strain in real time, while titrating positive end-expiratory pressure (PEEP) to minimize dynamic lung strain with an electrical impedance tomography belt. The ventilator screen displays a single-breath 10-cmH_2_O PEEP reduction used to perform the recruitment-to-inflation ratio maneuver. The presence of overdistension during tidal ventilation is also assessed by evaluating respiratory mechanics after the application of an abdominal weight. An esophageal catheter is used for estimating patient’s inspiratory effort and transpulmonary driving pressure during partial assist ventilator support. This image is illustrative. Many of the described techniques still require rigorous physiological and clinical validation before adoption into routine bedside practice.

Mechanical ventilation, while lifesaving, can also cause lung injury, an idea recognized as early as the 18th century and now referred to as VILI. Modern understanding of VILI focuses on lung stress and strain, which are influenced by factors such as Vt, lung compliance, airway pressure, and inspiratory muscle pressure. Timely assessment of lung overdistension is critical for optimizing ventilatory support and several innovative techniques have been investigated in recent years for this purpose. While an expanding array of tools is enriching the armamentarium of the modern intensivist for detecting lung overdistension (Figure 1), most still await rigorous physiological and clinical validation before they can reliably inform real-time, bedside assessments of stress and strain.
